# Improving Quality of Preventive Care at a Student-Run Free Clinic

**DOI:** 10.1371/journal.pone.0081441

**Published:** 2013-11-21

**Authors:** Neel M. Butala, Harry Chang, Leora I. Horwitz, Mary Bartlett, Peter Ellis

**Affiliations:** 1 Yale University School of Medicine, New Haven, Connecticut, United States of America; 2 Yale University School of Medicine, Department of Internal Medicine, New Haven, Connecticut, United States of America; 3 Fair Haven Community Health Center, New Haven, Connecticut, United States of America; Aga Khan University, Pakistan

## Abstract

Student-run clinics increasingly serve as primary care providers for patients of lower socioeconomic status, but studies show that quality of care at student-run clinics has room for improvement.

**Purpose:**

To examine change in provision of preventive services in a student-run free clinic after implementation of a student-led QI intervention involving prompting.

**Method:**

Review of patient charts pre- and post-intervention, examining adherence to screening guidelines for diabetes, dyslipidemia, HIV, and cervical cancer.

**Results:**

Adherence to guidelines among eligible patients increased after intervention in 3 of 4 services examined. Receipt of HIV testing increased from 33% (80/240) to 48% (74/154; p = 0.004), fasting lipid panel increased from 53% (46/86) to 72% (38/53; p = 0.033), and fasting blood glucose increased from 59% (27/46) to 82% (18/22; p = 0.059).

**Conclusions:**

This student-run free clinic implemented a student-led QI intervention that increased provision of prevention. Such a model for QI could extend to other student-run clinics nationally.

## Introduction

Clinical preventive services, such as disease screening and age-appropriate physical examinations, are essential to improving health and have become a national priority[1]. Organizations such as the United States Preventive Services Task Force (USPSTF) publish guidelines on recommended preventive screenings to set standards for preventive care [2]. However, adherence to these guidelines remains a challenge in many primary care settings [3], [4], particularly those that provide care to low-income patients [5].

Student-run free clinics frequently serve as primary care providers for patients of lower socioeconomic status. Several studies have examined the quality of preventive services in student-run free clinics, and all have demonstrated room for improvement [6]–[10]. Thus, it becomes important to examine the effectiveness of strategies for quality improvement (QI) of preventive care in student-run free clinics.

Furthermore, student-run free clinics provide an important venue for experiential education in QI. The American Association of Medical Colleges endorses QI education in medical schools [11], but there are few published medical school QI curricula[12]. While some medical schools teach formal QI curricula and related skills-building, involving students actively in real-life QI projects is challenging to implement due to competing time demands with other educational goals [13]. Given the proliferation of student-run free clinics at medical schools over the past decade[14], these settings present an important educational opportunity for students to learn real-life QI skills and to impact the quality of care provided to a large, underserved population.

The HAVEN Free Clinic, a student-run free clinic affiliated with our home university and a local community health center, measured adherence to guidelines for preventive care in 2009 [10]. Our clinic found that rates of screening for HIV testing, fasting lipid panel, fasting blood glucose, and Pap smear were on par with rates nationwide, but fell short of national goals. As a result, student leaders implemented an intervention to improve adherence to guidelines for our patients for these four services.

This study seeks to evaluate whether a student-led QI intervention increased adherence to guidelines for four key preventive health services over two years. The findings from this study would demonstrate whether the student-run free clinic is a feasible venue for student-led QI and, if so, could serve as a model for improving adherence to preventive care guidelines in other student-run free clinics nationally.

## Materials and Methods

### Ethics statement

Our study was approved by the Yale University Human Investigations Committee, and we received a Health Insurance Portability and Accountability Act (HIPAA) waiver of informed consent.

### Setting

HAVEN operates on Saturdays and provides comprehensive primary care to uninsured adults living in the Fair Haven neighborhood of New Haven, CT. Additionally, we provide a wide range of other free services, including sub-specialty clinic days and referrals, social services, patient education, women's health services, support groups, and fitness classes. Many of the patients are immigrants to the U.S. and have not had medical care in several years. In 2012, HAVEN conducted 1066 patient visits and saw a total of 322 unique patients, averaging 24 unique patients per week.

Clinical teams at HAVEN consist of a senior clinical health professions student team member (SCTM), a junior pre-clinical health professions student team member (JCTM), and an interpreter, as necessary. Clinical teams work under the supervision of licensed attending clinicians. During this study period, HAVEN used paper-based records.

### Intervention

In 2009, HAVEN analyzed its rates of adherence to national preventive service guidelines for HIV testing, fasting lipid panel, fasting blood glucose, and Pap smear[2], [15]. We found that, while rates of provision for HAVEN patients were on par with rates of provision for individuals nationwide, HAVEN's rates were below those specified by national goals[10].

In response, HAVEN created a role for a new volunteer position, the Medical Records Specialist (MRS) in January of 2010. The MRS is a pre-clinical health professions student that reviews the charts of patients with upcoming appointments and notes any indicated preventive health screenings, vaccinations, or other follow-up items that were not addressed from previous visits. The MRS writes an “MRS Note” with checkboxes for each of these follow-up items in the paper chart directly adjacent to the physical space where the next clinical team would begin their note the following week. Thus, the clinical team would always be aware of recommended preventive screening follow-up items prior to seeing each patient.

The new MRS role was approved by the student leadership board and implemented in January 2010. After piloting the position for two trimesters, the position was subsequently combined with the JCTM position such that each JCTM volunteer would take one shift as an MRS per term.

## Method of evaluation

Annual chart review was used to evaluate the performance of the intervention. In 2008, the chart review was conducted on all charts from patients seen for a medical visit between October, 2007 and October, 2008. Starting in 2009, due to the growth in the clinic's patient panel, a simple random sample of charts from patients seen in the preceding year was selected. Starting in 2010, the sampling frame shifted from the end of October, 2009 to January 1^st^, 2010 to better align with clinic processes and interventions.

Demographic data was collected by preclinical volunteers and clinical data was collected by clinical health professions students on paper abstraction forms. Students received training beforehand and those supervising the chart review typically performed quality control checks by reviewing all charts with completed forms a second time. Data from the forms were transferred to an electronic database by preclinical volunteers.

Eligibility for preventive screening was determined according to 2009 USPSTF and American Diabetic Association guidelines on the basis of age and gender (Table 1). If patients were eligible in a particular year, we checked whether a screening was performed that year. Patients who had been screened in a previous year within the guideline-recommended window were not considered eligible. Observations with missing age or gender were excluded.

**Table 1:** Criteria & Guidelines for performance of preventive health services at HAVEN Free Clinic.

Preventive Health Service	Criteria for performance
HIV test*	Consider screening all patients once regardless of risk factors.
Fasting lipid panel*	Screen every 5 years for women aged 45 and over and men aged 35 and over; for women under age 45 and men under age 35, screen only if at increased risk for coronary heart disease.
Fasting blood glucose†	Screen every 3 years for those aged 45 and over; for those under age 45, only screen if at increased risk for insulin resistance.
Pap smear*	Screen every year for women aged 18–30 within 3 years of being sexually active or age 21 (whichever comes first); for those over age 30, screen every 3 years with 3 consecutive normal tests.
* US Preventive Task Force health maintenance guideline; † American Diabetic Association health maintenance guideline	

### Statistical Analysis

We divided the cohort into pre-observation (2008–2009) and post-observation (2010–2011) groups. We compared patient demographics in the pre and post-intervention periods using chi-square tests for categorical variables and student's t-tests for continuous variables. Additionally, chi-square tests were used to compare pre- and post-intervention proportions of eligible patients that had received a given screening in accordance with guidelines in a particular year. All analyses were conducted using SAS version 9.2 (SAS Institute, Cary, NC).

## Results

Of 493 charts reviewed, 24 had missing age or gender and were excluded from our analysis, bringing our final sample size to 469 (275 pre-intervention and 194 post-intervention). There were no significant differences in baseline characteristics in the sample before and after the intervention (Table 2).

**Table 2:** HAVEN Patient Demographic Characteristics.

Demographics	N (%)	Before Intervention n (%)	After Intervention n (%)	p-value
N	469	275 (59%)	194 (41%)	
Age (mean years)	35.8	36.3	35.1	0.264
Patient tenure				0.9378
*New*	248 (53%)	145 (53%)	103 (53%)	
*Returning*	221 (47%)	130 (47%)	91 (47%)	
Gender				0.064
*Male*	264 (56%)	145 (53%)	119 (61%)	
*Female*	205 (44%)	130 (47%)	75 (39%)	
Race/ethnicity				0.705
*Latino*	414 (88%)	240 (87%)	174 (90%)	
*Other*	46 (10%)	28 (10%)	18 (9%)	
Primary language				0.374
*Spanish*	393 (84%)	232 (84%)	161 (83%)	
*Other*	55 (12%)	29 (11%)	26 (13%)	
Highest education†				0.071
*Primary*	93 (31%)	30 (28%)	63 (32%)	
*Some secondary*	58 (19%)	13 (12%)	45 (23%)	
*Secondary/GED*	83 (28%)	28 (26%)	55 (28%)	
*Bachelor's and/or Graduate/Professional*	47 (16%)	22 (21%)	25 (13%)	
Employment status†				0.558
*Full time*	59 (20%)	23 (22%)	36 (19%)	
*Part time*	100 (33%)	34 (32%)	66 (34%)	
*Unemployed*	126 (42%)	39 (37%)	87 (45%)	
† 2009–2011 data only				

**Figure 1:**
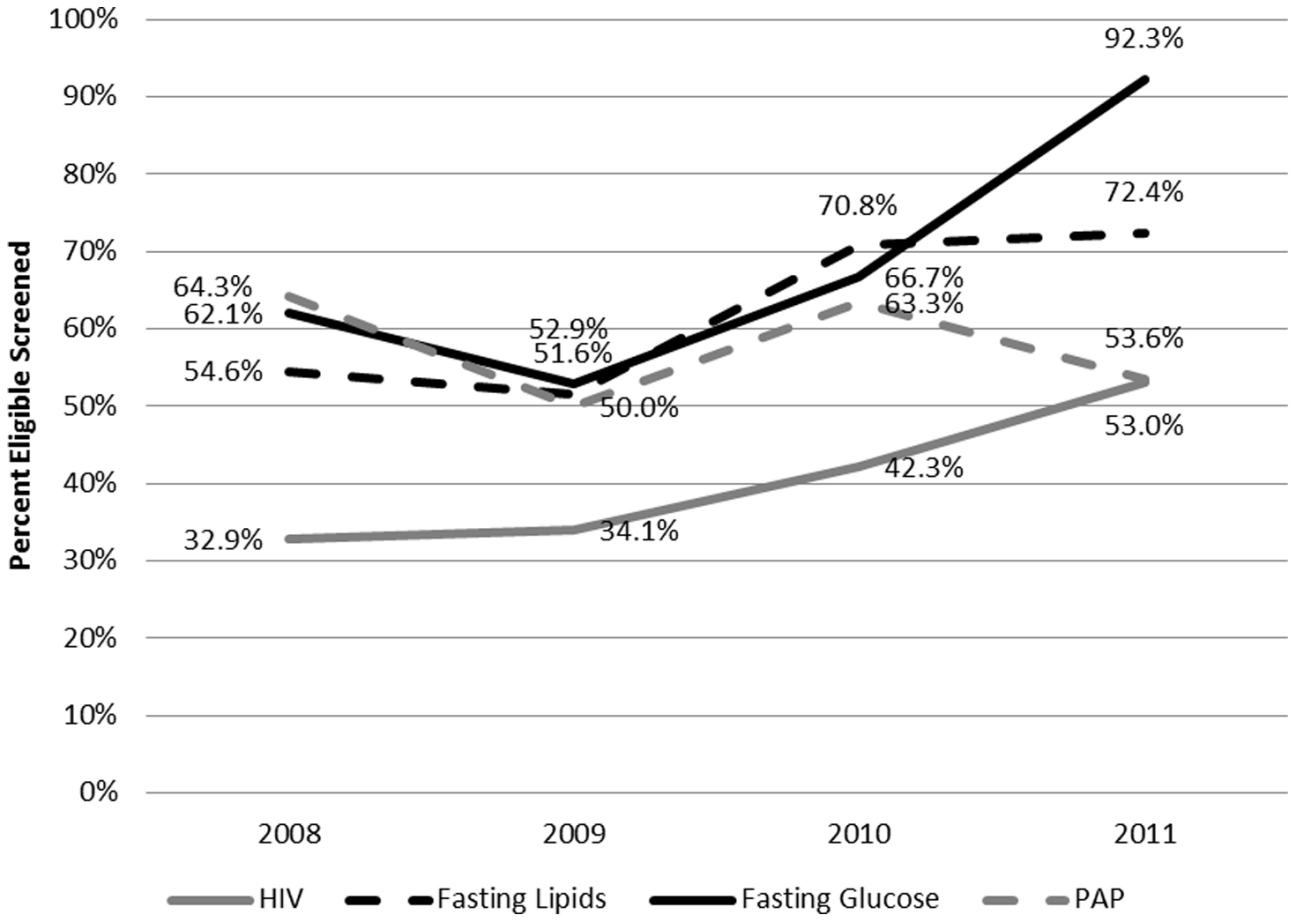
HAVEN Free Clinic Performance on Preventive Health Services over Time.

In all four measures of preventive screening, incident screening rates among those eligible for testing was equal to or higher after the addition of the MRS position (Figure 1, Table 3). Receipt of HIV testing increased significantly from 33% (80/240) of eligible patients pre-intervention to 48% (74/154; p = 0.0035) post-intervention. Similarly, receipt of fasting lipid panel increased significantly from 53% (46/86) of eligible patients pre-intervention to 72% (38/53; p = 0.0330) post-intervention. Receipt of fasting blood glucose screening increased from 59% (27/46) of eligible patients pre-intervention to 82% (18/22; p = 0.0594) post-intervention. Finally, receipt of Pap smear among eligible patients was constant at 59% (68/166 pre-intervention and 34/58 post-intervention).

**Table 3:** Comparison of HAVEN Free Clinic Performance on Selected Preventive Health Services Before and After Intervention.

Preventive Health Service	Pre-intervention n receiving/n eligible (%)	Post-intervention n receiving/n eligible (%)	P value
HIV test	80/240 (33%)	74/154 (48%)	0.0035
Fasting lipid panel	46/86 (53%)	38/53 (72%)	0.0330
Fasting blood glucose	27/46 (59%)	18/22 (82%)	0.0594
Pap smear	68/116 (59%)	34/58 (59%)	1.0000

## Discussion

Our study found that rates of guidelines-recommended screening increased for 3 out of 4 preventive care measures examined after a student-led quality improvement intervention in our student-run free clinic. We believe the primary driver of this change was the effective implementation of an MRS role to prompt clinical teams to consider preventive screenings. Through development and refinement of this intervention, health professions students gained an experiential education in QI.

The increased rates of HIV testing and cholesterol screening in our study are comparable to those after QI interventions in other primary care settings. For example, introducing a rapid testing protocol in six community health centers showed that HIV testing rates improved from 3% to 19% of the eligible population [16]. Similarly, after implementing computer-generated patient and physician reminders, cholesterol screening rates in a university-based family practice increased from 19.5% to 38.1% [17]. The gains reported in both of these studies are comparable to the 15% and 19% increases in HIV testing and cholesterol screening in our study.

Although we did not find an increase in Pap smear after our intervention, this lack of improvement is consistent with previous studies examining provider-targeted interventions for cervical cancer screening. A systematic review of interventions to increase Pap smear rates found that while patient-focused interventions tended to be effective, the impact of provider-focused interventions was heterogeneous and only marginally effective at best [18]. This finding suggests that many of the barriers to cervical cancer screening are patient-related, such as lack of education, forgetfulness, and fear of the test or disease itself [19], [20]. Nevertheless, there were two provider-centric factors that could have also affected our results. First, the guidelines for Pap smear changed over the period of study, and, at times, national guidelines were different from clinic guidelines, which may have contributed to confusion for clinical teams and have biased our results towards the null. Secondly, the invasive nature of the test may have made students or supervising clinicians who may not regularly perform gynecological exams uncomfortable with conducting a Pap smear when patients come in specifically for other acute complaints. While there is certainly a role for provider-focused interventions to improve cervical cancer screening in our clinic, future interventions should also focus on patient education and outreach.

We believe the change in preventive screening rates in our study is driven by the addition of an MRS role. The MRS note functions as a prompt that enhances the salience of preventive screening for the clinical teams who may otherwise be focused on acute issues. Prompts have been shown to improve adherence to guideline-recommended therapy in primary care settings in systematic reviews [21], [22]. We suspect that prompts for preventive screening may be even more effective in a student-run free clinic setting, where the dual and often competing goals of education and clinical service in the setting of resource constraints may limit the time of student-clinicians and divert their attention towards more acute priorities.

The findings of this study should be interpreted in light of its limitations. The observational pre/post design cannot establish a causal relationship between the implementation of the MRS position and the increase in prevention. However, the sharp sustained uptick in provision in the period after the inclusion of the MRS role, following relatively flat rates preceding the intervention, and a lack of change in clinic demographics is strongly suggestive of cause. Additionally, using age or gender alone may not capture some patients who have risk factors that that make them eligible for certain screenings at younger ages. However, we have no reason to believe that screening for such individuals would be differentially affected by our intervention and thus their exclusion likely would not bias our results. Finally, because data were combined from multiple years, patients may have repeated observations in our sample. However, this is unlikely to bias our results, as the proportion of returning patients each year was similar.

Considerations for future study include determining whether our approach can be amenable to increasing rates of other types of screenings or preventive interventions, such as vaccinations or domestic violence or alcohol abuse screening. Additionally, it would be important to identify if there is an upper limit on how many screenings an MRS can recommend at one time before adherence by clinical teams would start to decline. Finally, having identified a student-run free clinic as an effective venue for experiential QI education, it becomes important to quantify the educational impacts of such QI interventions in the future.

In conclusion, this study found that a student-led QI intervention can increase rates of preventive care in a student-run free clinic. The effectiveness of this intervention validates the venue of a student-run free clinic as a setting for experiential education in QI and student-led quality improvements. We believe the model for increasing adherence to preventive care guidelines presented in this study can extend to student-run clinics nationally.
